# Preliminary Study of Lead-Contaminated Drinking Water in Public Parks—An Assessment of Equity and Exposure Risks in Two Texas Communities

**DOI:** 10.3390/ijerph18126443

**Published:** 2021-06-14

**Authors:** Leanne Fawkes, Garett Sansom

**Affiliations:** Department of Environmental and Occupational Health, School of Public Health, Texas A&M University, College Station, TX 77843, USA; Leanne.fawkes@tamu.edu

**Keywords:** drinking water, lead, parks, environmental health

## Abstract

Safe drinking water is celebrated as a public health achievement and is a top priority for the Environmental Protection Agency. Yet today, lead (Pb) contaminated drinking water has the potential to be a public health crisis in the United States. Despite efforts to provide safe drinking water, update water infrastructure, and ensure strict drinking water regulations, there are incidents of unsafe lead levels and reports of associated adverse health effects. While there has been increased attention paid to the quality of drinking water within individuals’ homes, little research has examined the presence and concentration of lead in water from drinking fountain sources located in public parks. In this study, we sampled drinking water from every accessible public park in the Bryan/College Station (BCS), TX metropolitan area (*N* = 56). With a lower detection level of 2.0 μg/L, we discovered a mean lead concentration of 1.3 μg/L across all sites and a maximum of 8.0 μg/L. Furthermore, neighborhoods below the median income for BCS were twice as likely to have detectable lead levels in their water and had 1.5 times the mean concentration. This study underscores the need for action and supports previous studies that have identified a disparate burden to lead exposure among low socioeconomic populations within the United States. By examining the water quality in drinking fountains in publicly accessible parks, the results of our study provide public health professionals with important information about where infrastructure should be improved and the potential harms of lead in drinking fountain water.

## 1. Introduction

Contamination of drinking water by lead is a growing concern within the United States as the breadth of exposure has become clearer over the last decade. Contact with lead is associated with several adverse health outcomes and is particularly concerning for children, as no safe blood lead threshold has been identified [1,2]. Prior research has also conclusively shown that minority and low-income communities shoulder the bulk of lead exposure as compared with their more affluent counterparts [3]. The causal link between lead exposure and severe cognitive health effects has been well established as have other outcomes such as memory loss, abdominal pain, kidney damage, high blood pressure, and weakness [4,5]. There is a critical need to better characterize the presence and concentration of lead, especially within vulnerable communities.

Despite lead occurring naturally, there are no known safe levels of exposure in the environment [6,7]. In recognition of this, the maximum contaminant level goal (MCLG), set by the United States Environmental Protection Agency (EPA), is zero, as even trace amounts and low exposure levels are dangerous [8]. In spite of the health consequences of exposure, the EPA has established an action level for lead in drinking water of 15 μg/L. The action level is not designed to be a measure of health risks, but instead as a potential point at which additional action should be taken. The origin and concentration of lead exposure varies within differing groups.

The principal route of lead absorption is through the respiratory and gastrointestinal tracts; identifying the exposure route is imperative because it determines the lead absorption rate [9,10,11]. Inhalation of lead arises from breathing lead-contaminated dust, whereas ingestion of lead occurs by consuming contaminated drinking water, soil, or lead paint chips. An alternative route of lead exposure to the human body is dermal absorption; this is likely to occur when an individual comes into contact with lead-contaminated surfaces. Exposures for those under five years old and developing children are of principal concern. According to body weight, children breathe more air and consume more water than adults; therefore, this means they are likely exposed to higher environmental contaminants. Whereas adults 20 years old and older consume 17 mL/kg/day of tap water, children younger than six months consume 88 mL/kg/day [12]. Additionally, young children explore their environments and learn by engaging in hand-to-mouth and object-to-mouth behaviors [12]. In children, lead exposure can lead to numerous health effects, including low development, cognitive impairments, encephalopathy, renal effects, and developmental problems for their offspring [6]. While exposure may occur through a variety of sources, drinking water encompasses a large portion of a person’s total risk.

Some work has been accomplished assessing the presence and concentration of lead in public spaces. For instance, a study by Bryant (2004) [13] found more than half of Philadelphia’s public-school buildings had lead levels exceeding the EPA’s action level of 20 ppb. Furthermore, a study assessing drinking fountains in large public offices revealed that there were traces of lead detected in 37.5% and copper in 100% of tested water fountains (*n* = 45) [14]. There has been some movement in assessing public drinking fountains, for instance, the New York City Parks Department began a multi-year project, in 2019, to sample every park’s drinking fountain for the presence and concentration of lead; currently, over 250 fountains have been found in excess of the EPA’s action level [15].

Detectable traces of lead in drinking water frequently arise from lead pipes, faucets, and fixtures [5]. Cities and homes are more likely to have lead pipes if they were built before the 1970s, although some homes built as late as 1986 may have lead pipes due to antiquated plumbing infrastructure [9]. Most U.S. cities ceased using lead water pipes in the 1920s; this was not unanimous, however, until the national model plumbing codes were established. Studies have estimated that millions of U.S. households receive water from full or partial lead pipe systems and approximately 22 million people drink from them [14]. Monitoring existing pipe networks is vital for water managers because changing the water chemistry can impact the exposure risks [16]. Lead pipes concomitant materials can leave a hazardous multigenerational legacy; therefore, proper oversight and maintenance cannot be overstated. The magnitude of the problem is challenging to estimate due to under-reporting, minimal screening, and other related factors. Yet, some researchers estimate that approximately 1.2 million children had elevated blood lead levels (EBLL) from 1999 to 2010 and only half (606,709) were reported to the Centers for Disease Control and Prevention (CDC) [17].

Historically, populations most at risk for lead exposure in the United States are children from families living at or below the federal poverty level and ethnic minority groups. Poverty is connected to minimal access to resources, low-quality physical environments, and risk factors associated with substandard housing. Risks of living in substandard housing include the home’s age, the presence of lead-based paint, lead plumbing, and contaminated soil [18,19]. Incidents such as the public health water crisis in Washington, DC (2001–2004), and Flint, Michigan (2014–2016) highlighted the need for continuous water quality management, infrastructure upkeep, and surveillance of lead exposures from water in the United States [20]. The 2018 United States Environmental Protection Agency (EPA) Drinking Water Infrastructure Needs Assessment indicates that maintaining and improving the nation’s drinking water infrastructure is a costly endeavor and requires approximately 472.6 billion USD over 20 years for improvements.

While this renewed interest in assessing for the presence and concentration of lead within individuals’ homes is an excellent step towards improving public health measures, little research has attempted to characterize potential lead exposure from public drinking fountains and no data is available for point source detection within our research site. In this study, we sampled drinking water from every accessible public park in the Bryan/College Station (BCS) metropolitan areas (30.8251° N, 96.4930° W) located in Texas. We used the data to test the hypothesis that the existence of lead contamination was associated with older infrastructure as well as lower-income neighborhoods. The high cost of sustaining publicly accessible drinking water fountains or water infrastructure should not outweigh the costs of potentially harming the nation’s children and local communities. In addition, to address potential health issues an understanding of the water conditions needs to be established.

## 2. Materials and Methods

### 2.1. Study Location

The study area of BCS is located in east-central Texas in Brazos County. Although the two cities are geographically conjoined, they vary vastly in their sociodemographic composition and characteristics. Table 1 presents a comparative glance at the statistics for the study areas.

In contrast to the population of United States with a poverty estimate of 14.1% in 2018, Bryan/College Station’s unusually high share of people in poverty can be partly explained by the presence of a substantially large student population. While both cities are predominantly White, Bryan has a larger minority population than College Station. Bryan and College Station cities source their drinking water entirely from groundwater pumped from 10 wells in the Simsboro Aquifer and one well from the Sparta Aquifer [22]. The Simsboro Aquifer is a part of the major Carrizo-Wilcox Aquifer (66 counties). The water in Brazos County is marginal to moderately saline and has total dissolved solids that range from 1000 to 7000 milligrams per liter. In 2005 and 2006, the Texas Water Development Board analyzed over 300 groundwater samples for major and minor ions, trace elements, and radionuclides. Groundwater results revealed water that was generally suitable for human consumption with a small minority exceeding the maximum contaminant levels and secondary standards for nitrate, lead, fluoride, chloride, sulfate, iron, manganese, and dissolved solids [23]. A majority of the water sourced from Sparta is used for agricultural purposes. However, in Brazos County, this water is multipurpose and is used for municipal, industrial, and irrigation purposes.

The presence of lead in drinking water is most commonly due to using a combination of chloramines and chlorine during the purification process and lead in distribution lines or plumbing [24,25]. For disinfecting water, both Bryan and College Station disinfect their drinking water with gas chlorine to provide an effective chlorine residual [26,27]. As mentioned above, lead-bearing plumbing parts such as solder, galvanized piping, and “lead-free” brass fixtures (can contain up to 8% lead) can leach lead. Another contributing factor to lead in potable water is lead service lines (LSLs), which are found in an estimated 15–22 million homes built before lead abatement programs. This pipe material requires diligent oversight outlined in the Lead and Copper Rule, including minimizing corrosivity, monitoring high-risk homes, and educating the public about potential or actual dangers [28]. The cities of Bryan and College Station, Texas, mostly use soft copper or PVC service lines following Texas Commission on Environmental Quality regulations [29].

**Figure 1:**
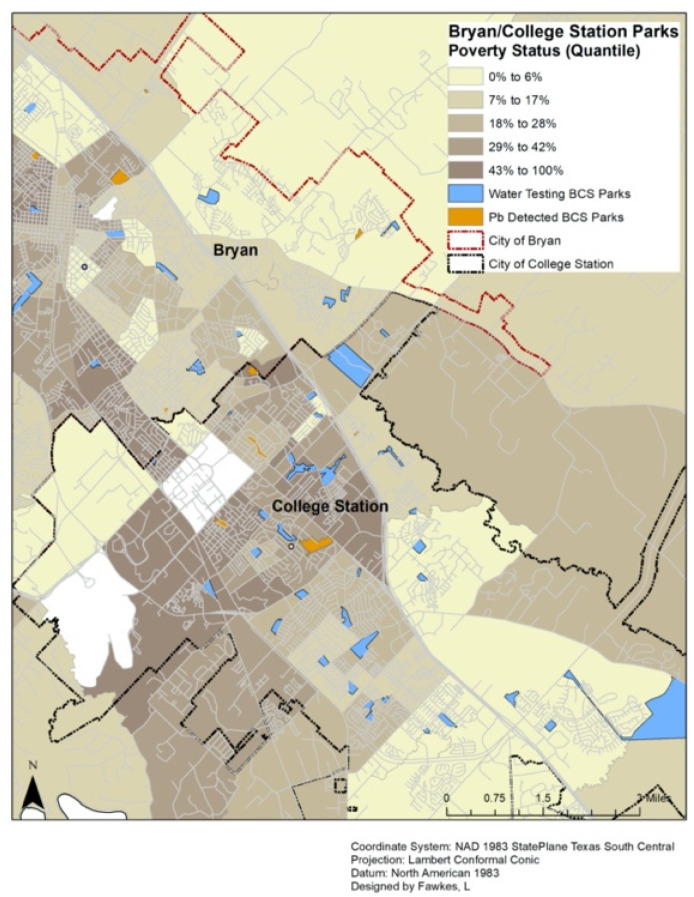
Location of Bryan and College Station, Texas parks by poverty status. Notes: BCS = Bryan/College Station; Pb = Lead.

Public parks were identified before sampling by using the BCS Parks and Recreation websites. [30,31] There were 56 parks listed on the website for the City of Bryan website; there were 54 parks recorded on the park registry for the City of College Station. Parks were excluded if they did not have a drinking fountain, were closed to the public, or their location was unidentifiable. All accessible parks with drinking fountains were included in this research. As a complete census of drinking fountains was sought for this research, no controls or differing selection criteria was used. Each area that was sampled can be seen in Figure 1 along with locations that had detectable lead levels. Furthermore, the percent of the community that is below the poverty level is shown in five quantiles.

### 2.2. Drinking Water Sampling

In July and August of 2020, water samples were collected from drinking fountains in every public park containing a drinking fountain in BCS. Non-powdered disposable gloves were used to avoid cross-contamination. Approximately 5 mL of water was collected from each location. The water samples were obtained in the morning hours before noon in an attempt to be the first draw of the day. The water sample analysis was executed in the field using the Palintest SA 1100 Scanning Analyzer (Palintest, Erlanger, KY, USA). The Palintest SA 1100 Scanning Analyzer provides the only portable EPA approved method to assess lead in drinking water and has a lower limit detection of 2.0 μg/L and a precision of ≤1.0 μg/L [32].

## 3. Results

There were 56 locations that met the inclusion criteria of this study, among those 21.4% of fountains (*n* = 12) had detectable lead levels at or above 2 μg/L. With a lower detection level of 2.0 μg/L, this study discovered a mean lead concentration of 1.3 μg/L across all sites and a maximum of 8 μg/L. The Census ACS one-year survey reports that the median household income for the College Station/Bryan metro area is 55,670 USD [32]. Using this economic threshold, neighborhoods with a mean household income below this were twice as likely to have detectable lead levels in their parks as compared with neighborhoods with a higher socioeconomic status (SES).

**Figure 2:**
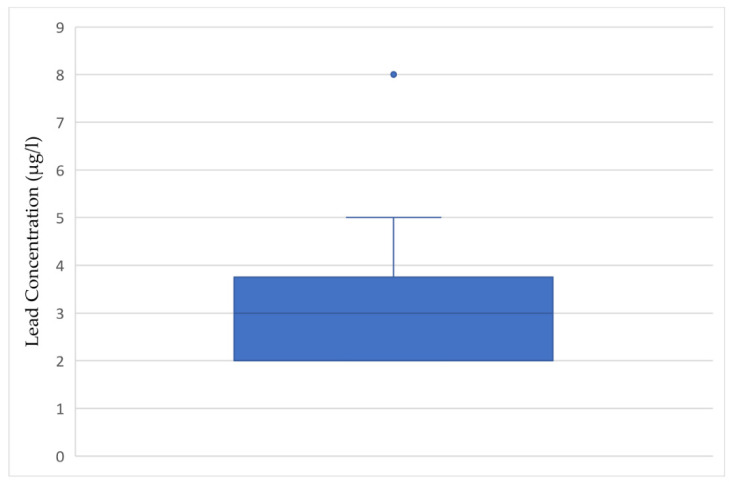
Box and whisker plot of concentrations (μg/L) of lead in water sampled from drinking fountains with detectable levels (*n* = 12).

As can be seen in Figure 2, the levels of lead in the samples did not exceed the EPA’s action level of 15 µg/L or the World Health Organizations level of 10 μg/L; however, all were above the maximum contaminant level goals. Of the parks that had detectable lead levels, there was a mean of 3.5 μg/L, with a median of 3 μg/L, and most findings were 3 µg/L, with one outlier at 8 μg/L, which was greater than twice the mean value detected. This figure reveals a wide spread of detected levels with a median of 3 µg/L.

## 4. Discussion

While there is growing attention being paid to the potentially inappropriate ways in which municipalities purify their drinking water, there remains a need to understand additional avenues of lead being introduced into our drinking water. There is a further need to better characterize all the potentially harmful exposures that can occur. Currently, the EPA has proposed revisions to the Lead and Copper Rule including a suite of actions to reduce lead exposures. This explicitly includes identifying the most impacted communities and protecting children from unnecessary exposures [33]. Our geographically compact preliminary study was designed to assess the existence and extent of lead contamination in water from public drinking fountains in BCS parks in response to these proposed changes. The findings from this study suggest that there is a risk associated with utilizing drinking fountains from publicly available parks and further points to a growing divide between low and high SES communities. A maximum of 8 μg/L was witnessed in one fountain that was in a park that was significantly older than other locations, which may account for the higher readings.

This study has several important limitations. The data was collected at one point in time at each location, and therefore may not represent an expected exposure from these sites. Furthermore, the lower detection limit was 2 μg/L, therefore, we were unable to detect trace amounts of contamination. Lastly, only one fountain was assessed per park, limiting the ability to make more inferences regarding the reason behind lead contamination. While children and families were seen playing at several of the parks, we did not assess how many were drinking from the fountains or using water from different sources. The strengths of this study include the complete ascertainment of parks in BCS and utilization of the only EPA approved portable lead analyzer.

Scholars continue to provide suggestions on removing lead from drinking water, by using activated carbon, mud, ash, clay, goethite, or soil [34,35]. Although these strategies address the problem after it has occurred, public health practitioners tend to intercede further upstream. The EPA recommends reducing exposure to lead contaminated drinking water by only using cold water for consumption and preparing baby formula, cleaning the faucet screen, using a certified water filter that removes lead, or running water before use. The other recommendation is to replace lead services lines, which can be cost prohibitive for some households because it is the responsibility of the utility service and homeowner.

## 5. Practical Implications

Future areas of research should include assessments of drinking fountains in other public parks and schools, especially if the study areas have children with known elevated blood lead levels. Parks and recreation departments should consider purchasing an EPA approved portable lead analyzer. Similar research has already begun to be conducted utilizing the same methods as this project in areas that children may consume water [36]. The consequences of not providing children in cities with safe lead-free drinking water are too great. The outcomes of this study provide information which enables policy makers an opportunity to improve the management of risks in parks and similar locations, where drinking water quality is not routinely monitored. On the basis of the findings of this study the cities of Bryan/College Station should replace or remove the drinking fountains with detectable lead levels and implement an improved monitoring program as witnessed in other locations [15].

## 6. Conclusions

Lead contaminated drinking water even at low levels is harmful to human health. Great strides toward the elimination of lead in drinking water have been made; however, approaching this problem at the local level may be the key to addressing the geographic areas of need of the most vulnerable populations. The findings of this study are noteworthy, because locating lead in over 20 percent of public parks in family-frequented venues is high as compared with other efforts that have looked at contamination in parks [15]. Moreover, an increase in actionable policies, population-specific interventions, and funding is needed to eradicate this deleterious public health problem. There is further cause for optimism within BCS, as the source of the lead is almost certainly from older infrastructure within the fountains themselves; there is a relatively inexpensive solution to ensure the health and wellbeing of community members. The findings of this research are underscored by the American Academy of Pediatrics (AAP) recommendation that drinking water does not exceed 1 μg/L lead for children [37]. In order to reach this goal a firm understanding of the current conditions must be understood

## Figures and Tables

**Table 1:** Sociodemographic characteristics for Bryan/College Station, TX.

Characteristics	Bryan	College Station
Total population	83,199	110,782
Population in poverty (%)	22.6	30.8
Population below 5 years of age (%)	7.5	5.3
Hispanic or Latino population (%)	39.5	15.6
Black or African American population (%)	15.8	7.4
Asian population (%)	1.9	10.4

Source: U.S. Census Bureau; 2018 ACS 5-year Estimates [21].

## Data Availability

Data is available for any reasonable request.
